# The impact of conjunctival flap method and drainage cannula diameter on bleb survival in the rabbit model

**DOI:** 10.1371/journal.pone.0196968

**Published:** 2018-05-24

**Authors:** Cooper D. Rodgers, Alissa M. Meyer, Nicole C. Rosenberg, Zachary L. Lukowski, Jamie L. Schaefer, Gina M. Martorana, Monica A. Levine, Craig A. Meyers, Mark B. Sherwood

**Affiliations:** Department of Ophthalmology, University of Florida, Gainesville, Florida, United States of America; University of Missouri-Columbia, UNITED STATES

## Abstract

**Purpose:**

To examine the effect of cannula diameter and conjunctival flap method on bleb survival in rabbits undergoing cannula-based glaucoma filtration surgery (GFS).

**Methods:**

Twelve New Zealand White rabbits underwent GFS in both eyes. The twenty-four eyes were divided into four groups. Two of the four groups (N = 12) received limbus-based conjunctival flaps (LBCF), and the other two (N = 12) received fornix-based conjunctival flaps (FBCF). Six FBCF rabbit eyes were implanted with 22-gauge drainage tubes, and the other six were implanted with 26-gauge tubes. Likewise, six LBCF rabbits received 22-gauge drainage tubes and six received 26-gauge tubes. Filtration blebs were evaluated every three days by a masked observer. Bleb failure was defined as the primary endpoint in this study and was recorded after two consecutive flat bleb evaluations.

**Results:**

Group 1 (LBCF, 22- gauge cannula) had a mean bleb survival time (Mean ± SD) of 18.7 ± 2.9 days. Group 2 (LBCF, 26-gauge cannula) also had a mean bleb survival time of 18.7 ± 2.9 days. Group 3 (FBCF, 22-gauge cannula) had a mean bleb survival time of 19.2 ± 3.8 days. Group 4 (FBCF, 26-gauge cannula) had a mean bleb survival time of 19.7 ± 4.1 days. A 2-way analysis of variance showed that neither surgical approach nor cannula gauge made a statistically significant difference in bleb survival time (*P* = 0.634 and *P* = 0.874). Additionally, there was no significant interaction between cannula gauge and conjunctival flap approach (*P* = 0.874), suggesting that there was not a combination of drainage gauge and conjunctival flap method that produced superior bleb survival.

**Conclusion:**

Limbus and fornix-based conjunctival flaps are equally effective in promoting bleb survival using both 22 and 26-gauge cannulas in the rabbit model. The 26-gauge drainage tube may be preferred because its smaller size facilitates the implantation process, reducing the risk of corneal contact.

## Introduction

Glaucoma is a leading cause of blindness throughout the world and is estimated to affect 76 million people by 2020 [1]. Although elevated intraocular pressure (IOP) is often associated with this disease, glaucoma is chiefly characterized by optic nerve deterioration and visual field loss. In the initial stages of treatment, glaucoma is generally managed with medication. When pharmaceuticals fail to reduce IOP to appropriate levels, trabeculectomy (glaucoma filtration surgery; GFS) and tube-shunt surgery are the two mainstay surgical procedures. The concept of the surgeries is similar: both reroute aqueous from the anterior chamber of the eye to the subconjunctival space, forming a filtration bleb. Although there is debate as to which is more efficacious, the success of both procedures is largely dependent on bleb formation and survival.

Surgical fashioning of a conjunctival flap is a key element of both GFS and tube-shunt surgery. This conjunctival flap can be either limbal-based (LBCF) in which an incision is made in the conjunctival and Tenon’s capsule tissues several millimeters behind the limbus or fornix-based (FBCF) in which the conjunctival and Tenon’s capsule incision is made at the limbus. Clinicians report several advantages and disadvantages to each surgical approach. While LBCFs are considered more difficult and time consuming to perform, some suggest that they have a lower risk of conjunctival wound leakage. In contrast, FBCFs are viewed as less technically demanding but with an increased risk of leakage [2].

Although the general trend has shifted towards the use of fornix-based flaps due to reports of increased rates of cystic blebs associated with LBCFs [3–5], a 2017 Cochrane systematic review found no significant differences in bleb survival or IOP control between FBCF and LBCF groups at 12 and 24 months follow-up in trabeculectomy patients. However, the group noted that LBCF eyes were more prone to be complicated by shallowing of the anterior chamber [6]. Compared to trabeculectomy, there is relatively little research regarding conjunctival flap method for tube-shunt surgery. One retrospective study by Suhr et al. found no significant differences in IOP control, overall success and changes in visual acuity between LBCF and FBCF tube-shunt eyes [7].

The rabbit model of GFS has been used to gain a better understanding of surgical techniques and has proven important in the development of drugs that reduce the likelihood of bleb failure, such as 5-flourouracil and mitomycin c [8, 9]. Initially, a full thickness sclerostomy was adapted for use in the rabbit model [10]. The sclerostomy model sometimes produced inconsistent results with not infrequent closure of the internal osteum by the iris or occasionally by vitreous. This lead to uncertainty in bleb survival endpoint determination, as scarring of the internal fistula is difficult to detect during clinical evaluation in this model. Later, Cordeiro et al. developed the 22-gauge angiocatheter model for rabbit GFS [11]. In this surgical procedure, a 22-gauge cannula is used to maintain a patent fistula between the anterior chamber and subconjunctival space. This method decreased the risk of internal occlusion by allowing the surgeon to place the tip of the cannula beyond the iris, in direct slit lamp view. The angiocath model of GFS produced more consistent results than sclerostomy, eliminating the uncertainty associated with internal occlusion.

Inserting the 22-gauge angiocath in a rabbit eye can prove difficult due to the shallowness of the rabbit anterior chamber (AC). The rabbit AC is 2.9 ± 0.36 mm deep on average compared to 3.5 ± 0.35 mm in humans [12]. Even when viscoelastic is used to deepen the AC, inserting the 22-gauge is challenging and may lead to significant peripheral iris and/or corneal contact. A smaller angiocath would be easier to insert and less likely to cause ocular damage. To our knowledge, there are no published studies analyzing the effect of drainage cannula diameter in the rabbit model of GFS.

Although it appears that the conjunctival flap method has little effect on long term IOP control, the effect on bleb survival in the rabbit model is yet to be determined. Here we analyze the effects of conjunctival flap method or GFS drainage cannula diameter on filtration bleb survival, using standard 22-gauge and smaller 26-gauge cannulas.

## Materials and methods

The study was performed using twelve New Zealand white rabbits, each weighing between 2kg and 4kg. The rabbits are sourced from Charles River Laboratories. The rabbits are housed in the AAALAC certified Animal Care Services facility at the University of Florida in Gainesville, Florida. The University of Florida Institutional Animal Care and Use Committee approved the experimental protocol prior to initiation of the study (study number- #201106599). Throughout the study, our protocol adhered to the Association for Research in Vision and Ophthalmology resolution statement for the use of animals in research.

### Study design

Twelve rabbits (a total of 24 eyes) were randomized to one of four treatment groups with six eyes in each group. All rabbits underwent glaucoma filtration surgery (GFS) in each eye from a single surgeon (MBS).

The four treatment groups were based on drainage tube gauge and conjunctival flap approach (Table 1):

**Table 1:** Experimental groups.

Group Number	N	Conjunctival Flap Method	Drainage Cannula Gauge
*Group 1*	6	Limbus-based	22
*Group 2*	6	Limbus-based	26
*Group 3*	6	Fornix-based	22
*Group 4*	6	Fornix-based	26

### Surgical operation

The rabbits were anesthetized using a combination intramuscular injection: 50mg/kg ketamine (“Ketaject”, Phoenix, MO) and 10/mg/kg xylazine (“Xyla-ject”, Phoenix, MO). Local anesthesia was also provided prior to surgery using topical administration of 0.1% proparacaine eye drops (Bausch & Lomb, Tampa, FL). The surgical technique used for the cannula-based glaucoma filtration surgeries was similar to those described in previous publications by this group [13–17]. All rabbits received the same surgical procedure aside from the conjunctival incision method and drainage cannula diameter.

In brief, the surgeon retracted the eyelids with the use of an eyelid speculum. A partial thickness corneal suture was then placed in the superior cornea as a traction suture, allowing rotation of the globe inferonasally. Surgical variations between experimental groups occurred at this step: Groups 1 and 2 (N = 12) received LBCFs, while Groups 3 and 4 (N = 12) received FBCFs.

For LBCF rabbits, Westcott scissors were used to make a posterior incision in the 6–7 mm from the limbus in the superotemporal quadrant. After the surgeon incised the conjunctiva, Tenon’s capsule was opened. The conjunctiva and Tenon’s capsule were then undermined toward the limbus taking care to not create any button holes in the superficial tissues.

In the FBCF rabbit groups, a standard 5 mm long incision was made at the limbus. The incision was extended around the limbus so that a scleral flap could be formed. Blunt dissection separated the conjunctiva from the Tenon’s capsule from the underlying sclera.

After the conjunctival flaps were fashioned, a #75 Beaver blade (Becton Dickinson & Co., Franklin Lakes, NJ) was used to form a corneal paracentesis tract in the superonasal quadrant and a cohesive viscoelastic agent was injected into the anterior chamber. Approximately 1 mm posterior to the limbus, a full thickness scleral tract through the anterior chamber was fashioned using a 27-gauge needle, taking care not to engage either the peripheral iris or cornea.

In twelve of the rabbits (six LBCF rabbits and six FBCF rabbits) a 22-gauge, IV cannula (Insyte Becton Dickinson Vascular Access, Sandy, UT) was inserted into the anterior chamber along the needle tract. A 26-gauge cannula was inserted in the other twelve (six LBCF rabbits and six FBCF rabbits). The needle of the cannula was retracted, and the cannula itself was placed inside of the pupillary margin to prevent occlusion by the iris. The scleral end of the drainage tube was trimmed so that it would protrude less than 1 mm from the insertion point. The cannula was anchored to the sclera using a 10–0 nylon suture (Ethicon Inc., Somerville, NJ).

In the FBCF group, Tenon’s capsule and the conjunctiva were closed in one layer using absorbable 8–0 polyglactin suture material (Vicryl ^®^, Ethicon Inc., Somerville, NJ) to form a watertight seal at the limbus. In the LBCF group a single layer running closure of the Tenon’s and conjunctiva was performed with the same 8–0 polyglactin (Vicryl ^®^, Ethicon Inc., Somerville, NJ) suture. After inflating the bleb with BSS via the AC paracentesis tract, a Seidel’s test was performed to check for bleb leakage. Following surgery, a topical ointment consisting of Neomycin and Dexamethasone was applied to control inflammation and prevent infection. Rabbits received an oral analgesic for two days post-operatively.

### Postoperative clinical evaluation

Post-surgically, rabbits were briefly anesthetized with isoflurane and examined by an experienced observer every three days. The observer assessed bleb elevation and area and evaluated the eyes for surgical complications such as hemorrhage, infection and shallowing of the anterior chamber. The bleb was judged flat when there was no separation of conjunctiva and Tenon’s tissues from the sclera and angiocath. After the observer judged the blebs to be flat on two consecutive occasions, bleb failure was recorded. The first of the two evaluation days where the bleb was recorded as flat was designated as the bleb endpoint. If the bleb was noted to be elevated after being declared flat on only 1 occasion, this time point was pre-determined in the study design not to count.

### Statistical analysis

A two-way analysis of variance (ANOVA) was performed using GraphPad Prism 5.0 software to examine the effect of cannula gauge and conjunctival flap method, as well as any interaction effects between the two methods. To detect a statistical power of 80% for either the 22 versus 26 or the fornix versus limbal based, there would have to be at least a 2.5 day difference in endpoint with this number of eyes.

## Results

Blebs in rabbits receiving LBCF survived an average of (Mean ± SD) 18.7 ± 2.9 days (Table 2). FBCF blebs survived fractionally longer at 19.4 ± 3.95, however this difference was not statistically significant (*P* = 0.634). No significant surgical complications were noted in any eye on day 1 or throughout the post-operative follow-up.

**Table 2:** Bleb survival of LBCF and FBCF rabbits using 22 and 26-gauge cannulas.

Conjunctival Flap Method	N	Average Bleb Survival Days Post-Op (Mean ± SD)	Tube Gauge	N	Average Bleb Survival Days Post-Op (Mean ± SD)
*Limbus-based*	12	18.7 ± 2.9	22	6	18.7 ± 2.9
			26	6	18.7 ± 2.9
*Fornix-based*	12	19.4 ± 3.95	22	6	19.2 ± 3.8
			26	6	19.7 ± 4.1

Eyes that were implanted with 22-gauge cannulas had an average bleb survival of 18.9 ± 3.4 days. This was not significantly different from 26-gauge eyes, which had an average bleb survival of 19.2 ± 3.6 days (*P* = 0.874).

**Fig 1:**
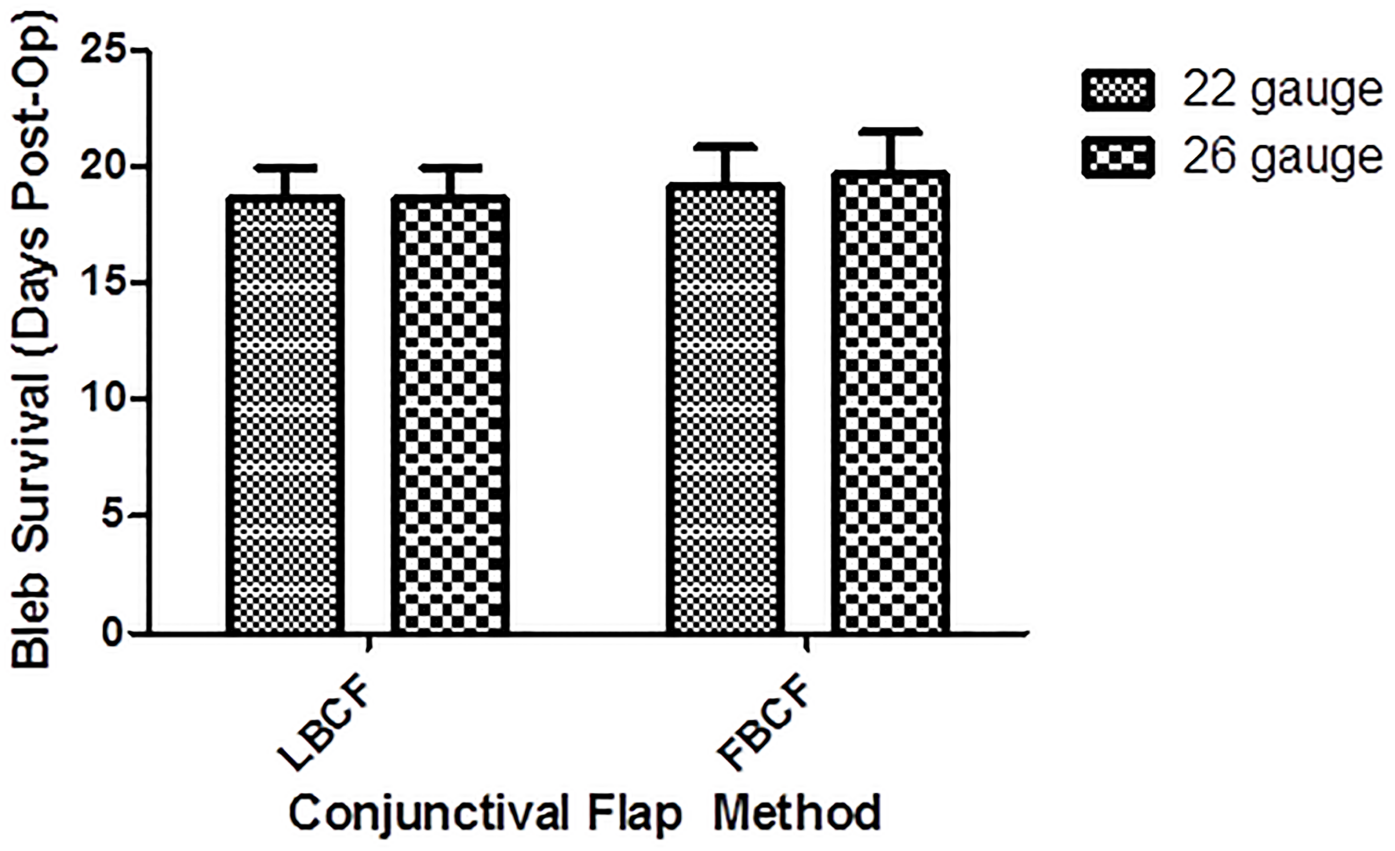
Bar chart showing the bleb survival of rabbits who received either limbus-based conjunctival flaps (LBCF) or fornix-based conjunctival flaps (FBCF) using either 22 or 26-gauge drainage cannulas. Bleb failure was declared after a masked evaluator deemed the bleb flat on two consecutive occasions.

**Fig 2:**
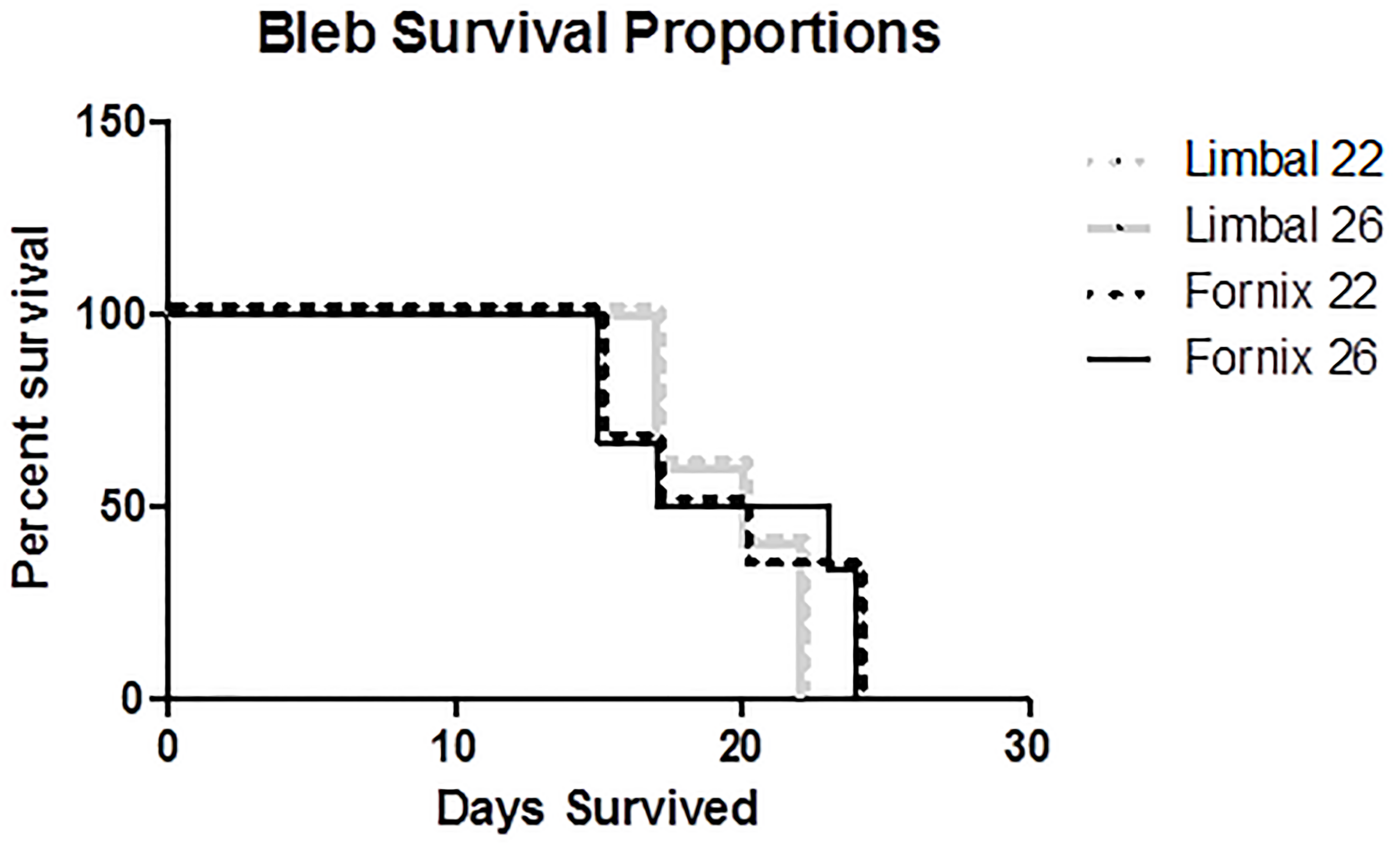
Kaplan-Meier bleb survival plot of rabbits who received either limbus-based conjunctival flaps (LBCF) or fornix-based conjunctival flaps (FBCF), using either 22 or 26-gauge drainage cannulas. Bleb failure was declared after a masked evaluator deemed the bleb flat on two consecutive occasions.

As depicted in Figs 1 and 2, LBCF rabbits had an average bleb survival of 18.7 ± 2.9 days when implanted with either 22 or 26-gauge drainage cannulas. Blebs in rabbits operated on with an FBCF approach survived an average of 19.2 ± 3.8 days when implanted with 22-gauge cannulas and 19.7 ± 4.1 with 26-gauge cannulas.

A two-way ANOVA revealed that there was no significant interaction between cannula gauge and surgical approach (*P* = 0.874), meaning that no particular combination of cannula gauge and conjunctival flap method produced significantly better survival results than the others.

## Discussion

Our study showed that conjunctival flap method did not have a significant impact on bleb survival in the rabbit model. The results of our study fall in line with previous retrospective human studies [6, 18–20] which showed no major differences in efficacy between the two methods. It appears that surgical skill and preference are the main factors that should determine conjunctival flap approach in the rabbit model.

In this study, bleb survival was chosen as the primary outcome measure rather than IOP. Intraocular pressure is known to be an unreliable indicator of bleb function in the rabbit model; there may be reductions in IOP even without patency between the AC and subconjunctival space [10]. This rabbit model is a model designed to study subconjunctival scarring, not glaucoma, as the aqueous outflow pathways are normal and baseline IOP is within the normal range. Therefore, bleb failure has generally been defined as the primary endpoint of GFS in the rabbit [21].

In 1973, Anthony Molteno developed the concept of draining aqueous away from the anterior chamber into a drainage plate via a long silicone drainage tube [22, 23]. Later in the early 1980’s, Stanley Schocket described another technique using readily available, inexpensive operating room materials. In this procedure, Schocket used an inverted retinal encircling band and 23-gauge Silastic tubing (N-5941-1, Storz) with an external diameter of 0.64 mm and an internal diameter of 0.34 mm to fashion a GDD [24, 25]. Since then, all of the more commonly used drainage implants including the Ahmed, Baerveldt and Molteno have adopted these tube dimensions.

Although 0.64 mm has been the default external drainage tube diameter for GDDs, there is a lack of research regarding alternative proportions. The cannula implanted during GFS can be considered a surrogate for the GDD drainage tube. The 22-gauge angiocath has a slightly larger diameter than the standard tube used in a GDD, but the 26-gauge is a smaller diameter. Usually, tube diameter does not present a problem for surgeons; humans have an AC that is sufficiently deep and can be easily expanded with viscoelastic solution. However, some patients have narrow drainage angles, particularly those with hypermetropia or those of East-Asian descent [26–28], where the iris is closer to the cornea, limiting space for tube placement.

Twenty-six and 22-gauge drainage cannulas were equally effective at promoting filtration bleb survival. Twenty-two gauge cannulas have an outer diameter of 0.67 mm, leaving a very small margin for insertion error. The American Academy of Ophthalmology has stated that corneal endothelial cell failure is the primary long-term problem associated with tube-shunt surgery [29]. Patients diagnosed with glaucoma may already have limited numbers of endothelial cells [30] and tube-endothelial contact may further damage the endothelium, leading to corneal edema and an increased likelihood of vision loss [31, 32]. The multicentered, prospective Tube versus Trabeculectomy study reported that persistent corneal edema was the most prevalent late post-operative complication associated with tube-shunt surgery, with 16% of tube eyes exhibiting this condition [33]. Similarly, the Ahmed versus Baerveldt study also reported a high rate of corneal complications with 11% of eyes complicated with persistent corneal edema [34].

Assuming an average rabbit AC depth of 2.9 ± 0.36 mm, there are between 0.95 and 1.31 millimeters of space on either side of the cannula once it is placed [12, 35]. The smaller 26-gauge cannula has an outer diameter of 0.404 mm, giving the surgeon improved clearance for implantation. A smaller diameter angiocatheter is easier to insert, decreasing the risk of complications from iris or corneal contact. Our results showed no statistically significant differences in bleb survival using 22 and 26-gauge drainage cannulas, suggesting that a 26-gauge drainage angiocatheter may be equally good for this glaucoma model and that GDD designers could consider using smaller gauge drainage tubes for patients.

In summary, drainage cannula diameter and conjunctival flap method produced no notable differences with respect to bleb survival in the rabbit model. The data presented support the use of 26-gauge cannulas in rabbit GFS in order to facilitate implantation and reduce post-operative complications. Further research is needed to examine the efficacy of smaller diameter GDD tubes in humans, especially in those patients with anatomically narrow drainage angles.

## Supporting information

S1 Table Bleb survival (days) and post-operative intraocular pressure after rabbit surgery. (XLSX)
